# Predicting the aggregation number of cationic surfactants based on ANN-QSAR modeling approaches: understanding the impact of molecular descriptors on aggregation numbers†

**DOI:** 10.1039/d2ra06064g

**Published:** 2022-11-24

**Authors:** Behnaz Abdous, S. Maryam Sajjadi, Ahmad Bagheri

**Affiliations:** Faculty of Chemistry, Semnan University Semnan Iran sajjadi@semnan.ac.ir +98-23-33384110 +98-23-31533192

## Abstract

In this work, a quantitative structure–activity relationship (QSAR) study is performed on some cationic surfactants to evaluate the relationship between the molecular structures of the compounds with their aggregation numbers (AGGNs) in aqueous solution at 25 °C. An artificial neural network (ANN) model is combined with the QSAR study to predict the aggregation number of the surfactants. In the ANN analysis, four out of more than 3000 molecular descriptors were used as input variables, and the complete set of 41 cationic surfactants was randomly divided into a training set of 29, a test set of 6, and a validation set of 6 molecules. After that, a multiple linear regression (MLR) analysis was utilized to build a linear model using the same descriptors and the results were compared statistically with those of the ANN analysis. The square of the correlation coefficient (*R*^2^) and root mean square error (RMSE) of the ANN and MLR models (for the whole data set) were 0.9392, 7.84, and 0.5010, 22.52, respectively. The results of the comparison revealed the efficiency of ANN in detecting a correlation between the molecular structure of surfactants and their AGGN values with a high predictive power due to the non-linearity in the studied data. Based on the ANN algorithm, the relative importance of the selected descriptors was computed and arranged in the following descending order: H-047 > ESpm12x > JGI6> Mor20p. Then, the QSAR data was interpreted and the impact of each descriptor on the AGGNs of the molecules were thoroughly discussed. The results showed there is a correlation between each selected descriptor and the AGGN values of the surfactants.

## Abstract 2

QSAR-ANN modelling was applied on some data obtained for surfactants to predict the aggregation number of the surfactants and conduct an explanatory study based the importance of selected descriptors.

## Graphical abstract

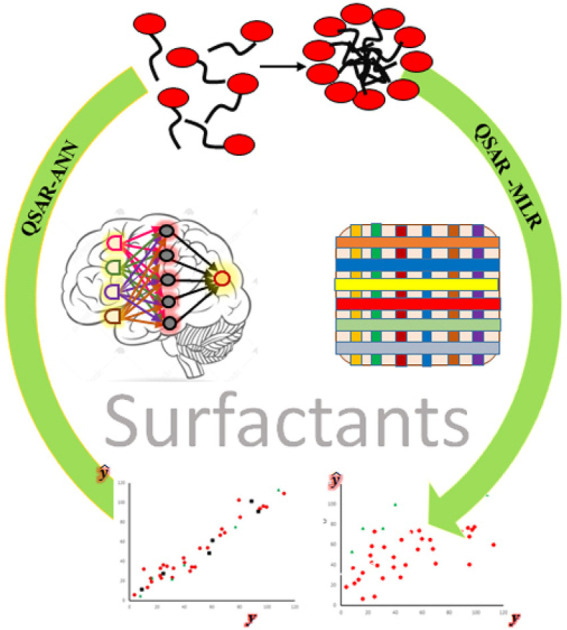

## 1. Introduction

Surfactants are among the most versatile chemical products and are widely used in the manufacture of cosmetics, detergents, pharmaceuticals, and in the textile industry, and so on.^1^ These materials have two main parts: a hydrophilic group (polar head) and a hydrophobic group (hydrocarbon chain). Based on the nature of the polar head, surfactants can be classified as: anionic, cationic, zwitterionic and non-ionic. Indeed, the amphiphilic structure of surfactants makes them highly suitable for surface activity. Among the surfactants, cationic molecules offer some additional advantages over the others. They show antibacterial properties apart from their surface, a fact which makes them applicable in the synthesis of cationic softeners, retarding agents, lubricants, and in some cases in consumer uses.^2–5^

The solution behaviors of cationic surfactants are commonly estimated using critical micelle concentration (CMC), aggregation number (AGGN) and degree of counter ion binding (*α*). The AGGN is the average number of surfactant molecules in a micelle unit and practically, the increase in AGGN leads to the formation of micelles which show great potential for use in many applications.^6^ For example, micelles with a greater AGGN have a greater capacity to transfer a drug in drug delivery systems or remove hydrocarbon contaminants in wastewater treatment processes.^7^ Therefore, measuring and establishing a AGGN is very significant.

There are versatile techniques to determine the AGGN of amphiphilic compounds including stepwise thinning of foam films,^8^ freezing point and vapor pressure methods,^9^ NMR spectroscopy,^10^ static light scattering,^11^ small-angle neutron scattering,^12^ small angle X-ray scattering,^13^ fluorescence probing methods,^14,15^ and electron paramagnetic resonance.^16^ Some of these are only applicable for AGGN determination at a surfactant concentration equal to CMC which only estimates the micelle AGGN for isolated non-interacting particles. In particular, the static light scattering method determines the AGGN values by calculating the molecular weight of the surfactant aggregate at the surfactant CMC. The static light scattering technique is rather complicated as it needs to determine the refractive index increment of the measured surfactant solution independently, and extrapolate the data to the CMC which does not let measuring the concentration dependence of the aggregation number. The small angle neutron scattering method allows the determination of the average micelle AGGN^12^ as well as providing information on the micelle shape. However, this technique is not easily available for a routine determination of micelle AGGN because of its complexity and the high cost of the neutron scattering experimental facilities.

Fluorescence probing strategies are commonly applied to estimate micelle AGGN where the estimation is influenced by neither the micellar shape nor by the interactions between the micelles. There are two types of fluorescence strategies: time-resolved fluorescence quenching (TRFQ) and a steady-state fluorescence method. The TRFQ technique calculates the micelle AGGN easily and accurately from the fluorescence decay curves.^17^ The steady-state fluorescence measurement has the benefits of conventional spectrophotometers, but it needs the application of single-photon counting equipment, and analysis by suitable non-linear fitting algorithms.

Overall, the fluorescent technique possesses the following advantages over the others: (i) it allows the quantification of AGGN at a given surfactant concentration, and in the presence of additives, (ii) it is not influenced by the phenomena of preferential adsorption, which greatly complicates the interpretation of the results, and (iii) it is applicable to all types of surfactants.^14,15^

The AGGNs of a large number of surfactants have been reported in the literature, based on fluorescence strategy, as these large volumes of data can be combined with modelling techniques to interpret the results, and can even be used to predict the AGGNs of new surfactants. Over the last 50 years, chemometrics has developed a powerful set of multivariate data modeling tools to help the “owner” of data find, plot and interpret statistically reliable patterns of data, and obtain maximal information from the studied system with minimal experimental effort.^18,19^

The QSAR modeling is one of the most versatile computational techniques for predicting the physical and biological properties of molecules, developed over the past decades. This technique has been widely recognized in a variety of fields such as medicinal chemistry, pharmacy, toxicology and material science.^20–22^ In fact, QSAR modelling can be used to find the relationship between the structure of chemical compounds, and their physical or biological properties to estimate the properties of new chemical compounds without the need for synthesis and testing. In QSAR analysis studies, molecular descriptors are numerical indices assigned to the molecular structure, and encode some information about the structure. Descriptors are theoretical indices which are computed by mathematical formulas or computational algorithms. The Dragon software is one of best, for finding the descriptors of a molecular structure; and it introduces a large variety of descriptors such as constitutional, topological and 3D-MoRSE descriptors, walk and path counts, and functional group counts.^23–28^

The predictive ability of a QSAR model is affected by the modeling techniques employed to find the mathematical model between the descriptors and their molecular activities. Basically, there are two general modelling methods used to analyze chemical science data, linear and non-linear. Linear approaches include MLR,^29^ principal component regression (PCR)^30^ and partial least-squares regression (PLS).^31^ Non-linear approaches include ANN,^32–35^ the support vector machine algorithm,^36^ the self-organizing map (SOM),^37^ radial basis functions neural networks (RBF),^38^ and multivariate adaptive regression splines.^39^

The ANN methods are known as non-linear learning math systems which construct a mapping of the input and output variables, and then the map is used to predict an unknown output as a function of suitable inputs.^32,40–42^ The main advantage of ANNs is that they can combine and incorporate both literature-based and experimental data to solve different problems such as predicting the toxicological and physical properties of surfactants.^43–45^

So far as is known, there is no report on predicting AGGNs of surfactants using linear or non-linear modeling techniques, and here, the non-linear ANN algorithm is proposed as a promising technique for this. A data set including 41 surfactant molecules was selected as a target study, and Dragon software was employed to compute the molecular descriptors of the surfactants and their experimental AGGNs were taken from previously published papers.^46–59^

In this study, firstly, the QSAR analyses of the surfactants were performed using MLR and ANN methods to compare the results of linear and non-linear models, and it was shown that the non-linear ANN model could find a satisfactory relationship between molecular descriptors and their AGGNs. Secondly, because the lengths of the hydrophobic group and polar head group are two important factors which strongly affect the AGGN,^55,56^ an explanatory study was conducted to interpret the impact of these factors on AGGNs based on the selected descriptor values such as H-047, ESpm12x, JGI6 and Mor20p.

## 2. Molecular database and software

In this study, a data set including 41 surfactant molecules was used and these structures are shown in Table 1. We took two points into account when collecting this data set: firstly, all the AGGNs of the molecules were obtained using the same strategy and experimental conditions. They were estimated by fluorescence decay curves of a micelle-solubilized pyrene method in aqueous solutions at 25 °C.^46–59^ Secondly, we struggled to collect the surfactant molecules with the same polar head group but a different length of the hydrophobic groups such as the following sets: (C_16_TAB, C_14_TAB, C_12_TAB, C_10_TAB, C_6_TAB); and ([C_16_MIM][Br], [C_14_MIM][Br], [C_12_MIM][Br], [C_10_MIM][Br], [C_9_MIM][Br]); and (C_16_E_2_TAB, C_14_E_2_TAB, C_12_E_2_TAB, C_10_E_2_TAB); and ([BisDec(MIM)_2_][2Br], [BisOct(MIM)_2_][2Br], [BisHex(MIM)_2_][2Br]). However, the structure of some molecules was only different in their polar head groups such as (C_16_TAB, [C_16_MIM][Br], CPC); and (C_12_TAB, [C_12_MIM][Br], C_12_DAB, DDMAC, DAC, DMAC).

**Table 1:** The molecular structure and AGGN value of each cationic surfactant used in the ANN-QSAR studies

No.	Symbol	Molecular structure	Predicted AGGN	Experimental AGGN	Set of data	Ref.
1	m-X-3	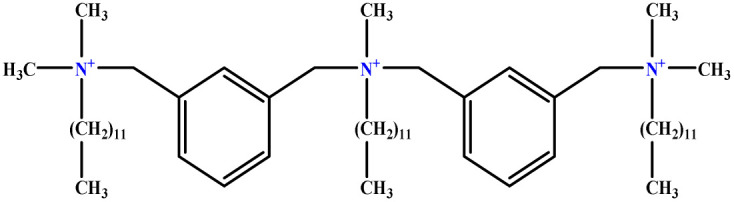	15.06	16	Training	46
2	EO-2	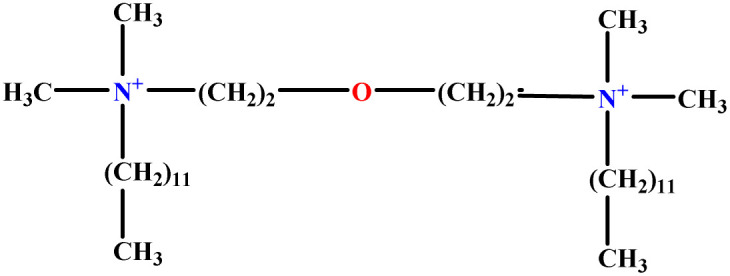	30.99	31	Validation	46
3	t-B-2	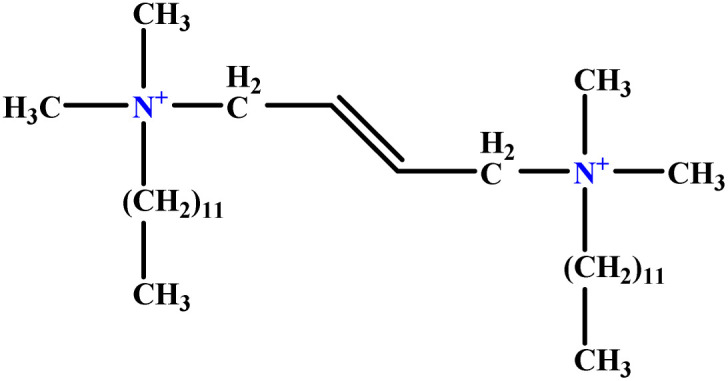	29.16	31	Training	46
4	o-X-2	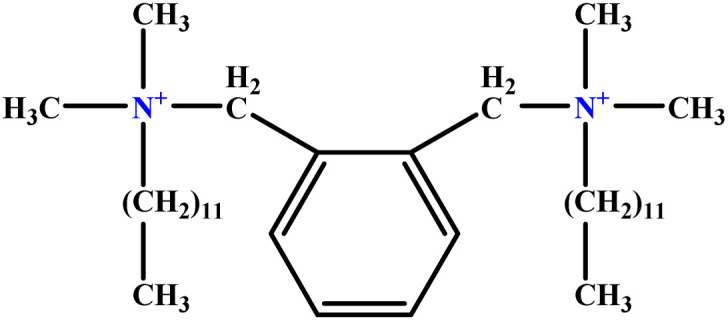	25.02	25	Test	46
5	BDDAC C_21_H_38_ClN	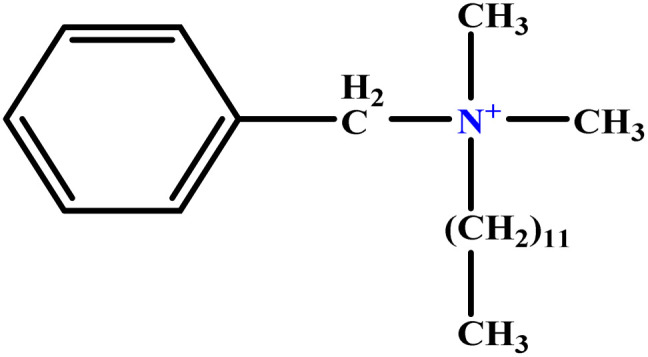	25.40	27	Training	46
6	[BisDec(MIM)_2_] [2Br]	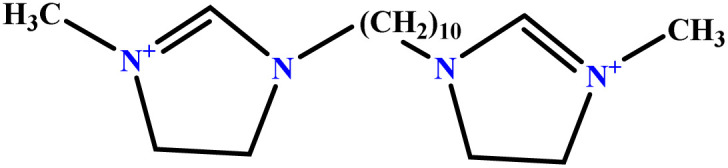	65.82	70	Training	47
7	[BisOct(MIM)_2_] [2Br]	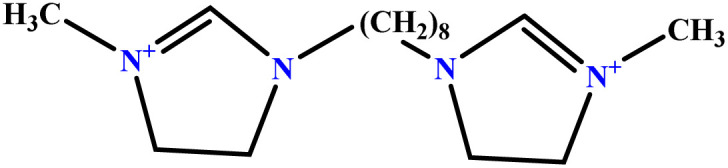	36.68	39	Training	47
8	[BisHex(MIM)_2_] [2Br]	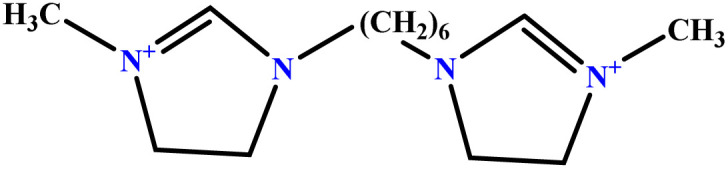	15.99	16	Validation	47
9	ValC3LS	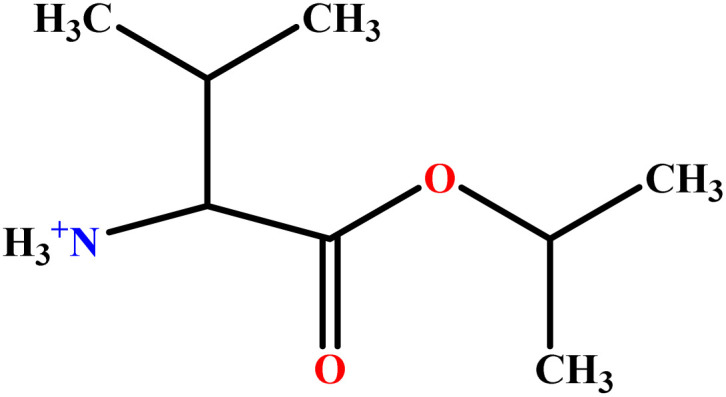	76.99	77	Validation	48
10	ProC3LS	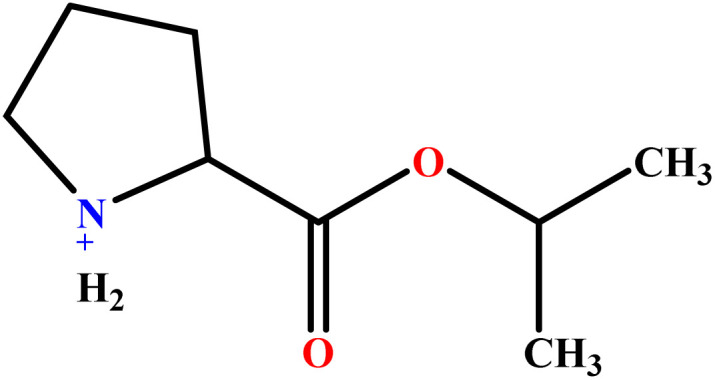	41.38	44	Training	48
11	AlaC3LS	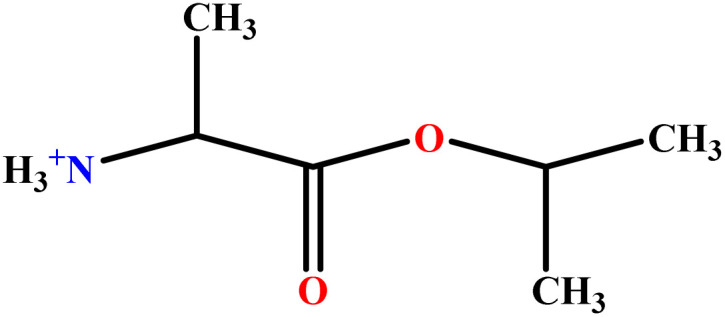	76.16	81	Training	48
12	GlyC3LS	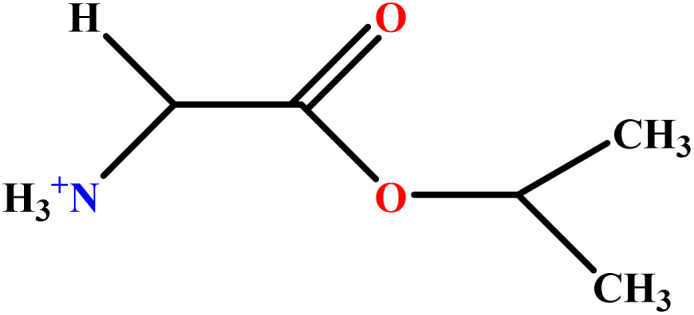	94.02	94	Test	48
13	[C_16_hpim]Br	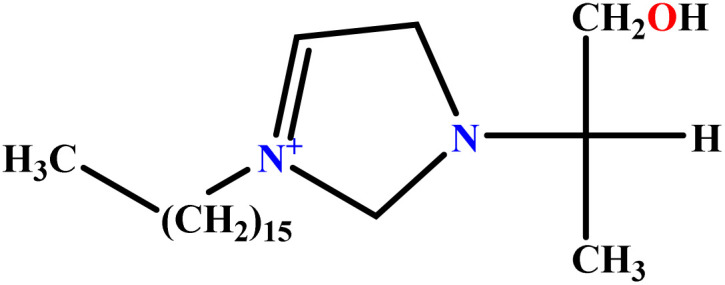	23.52	25	Training	49
14	L-UCPB	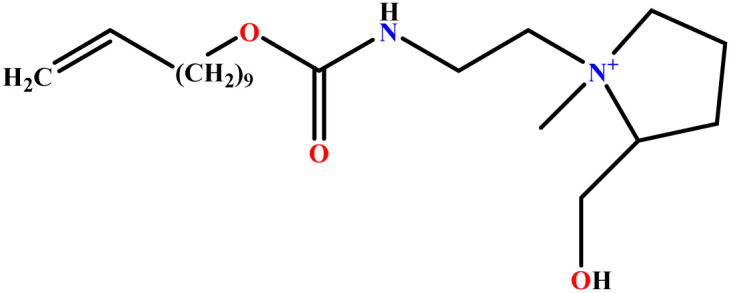	89.32	95	Training	50
15	LUCLB	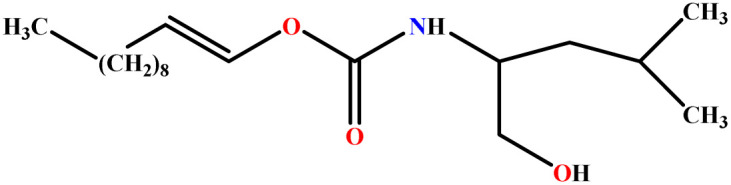	91.20	97	Training	50
16	[C_8_mim][Cl]	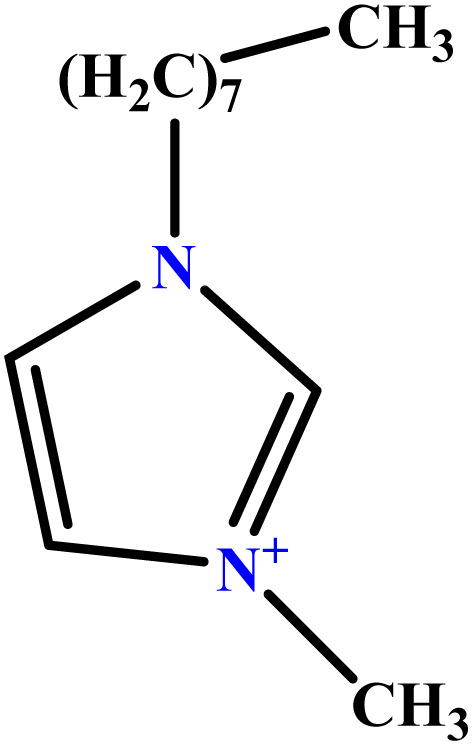	21.64	23	Training	51
17	[C_4_mpy][Cl]	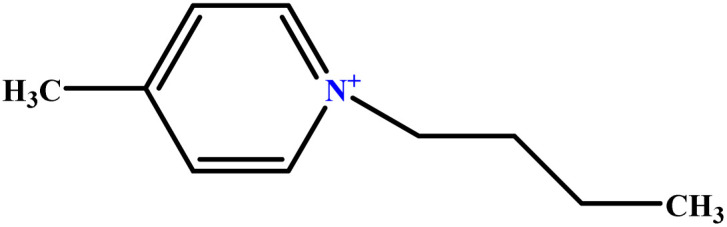	12.24	13	Training	51
18	[C_4_mim][Cl]	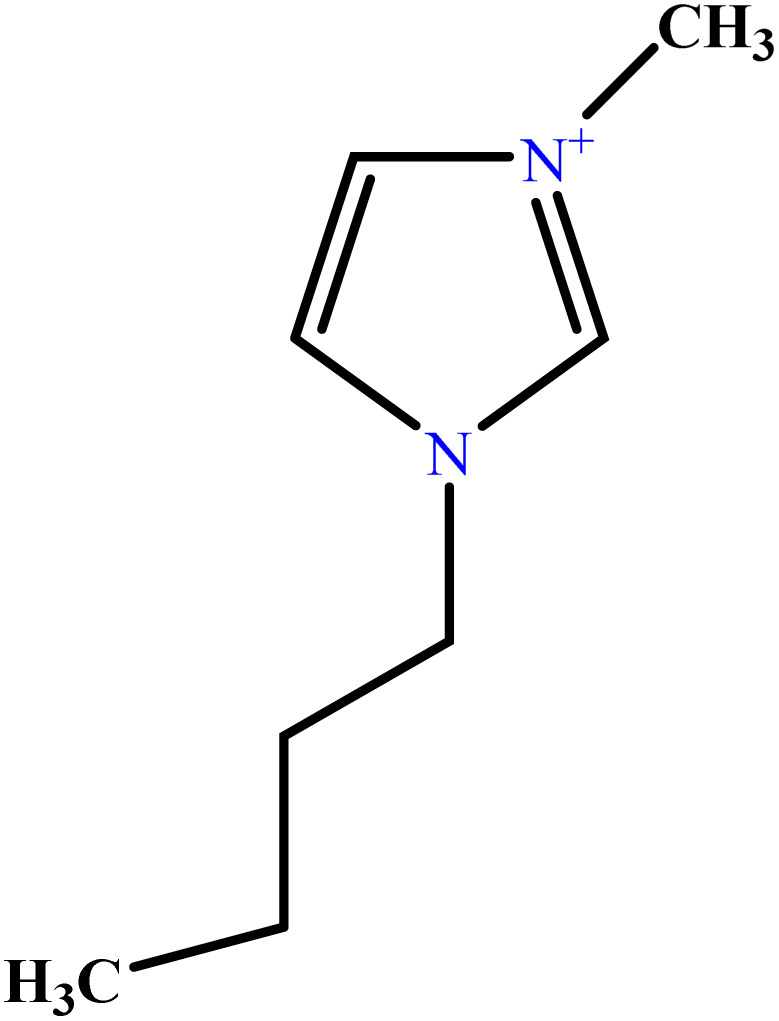	7.99	8	Validation	51
19	PH	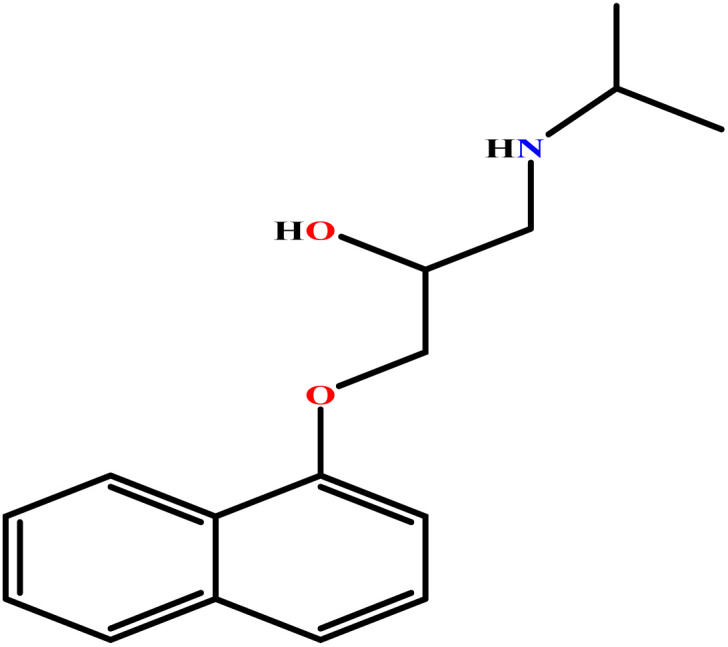	9.41	10	Training	52
20	C_16_E_2_TAB	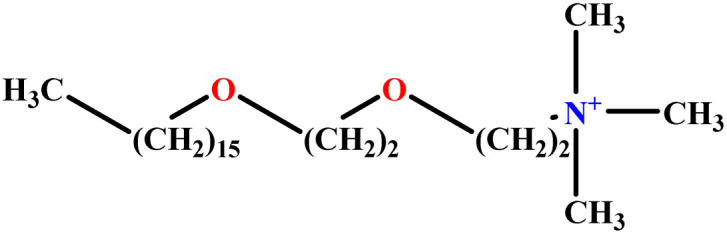	30.10	32	Training	53
21	C_14_E_2_TAB	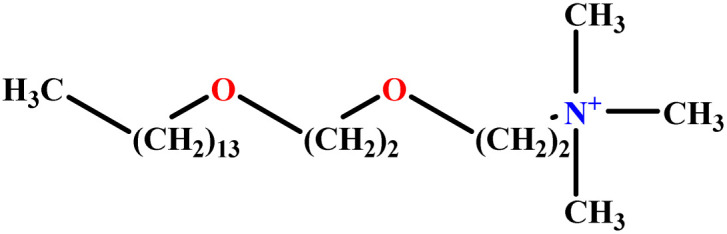	21.64	23	Training	53
22	C_10_E_2_TAB	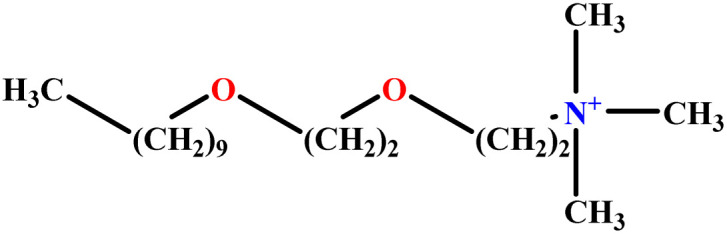	9.02	9	Test	53
23	C_12_E_3_TAB	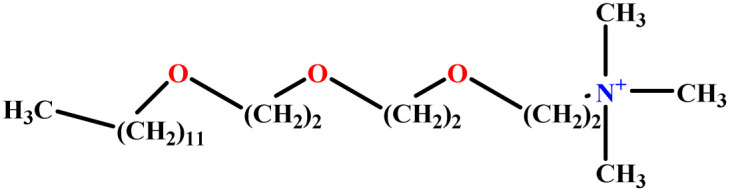	15.06	16	Training	53
24	C_12_E_2_TAB	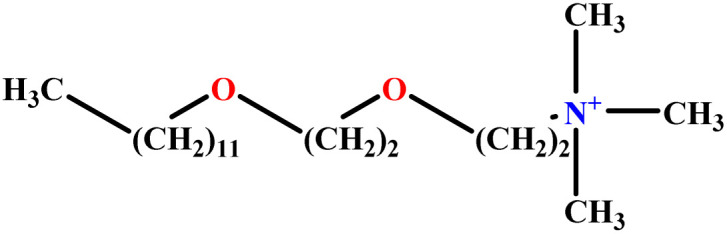	20.70	22	Training	53
25	DAC	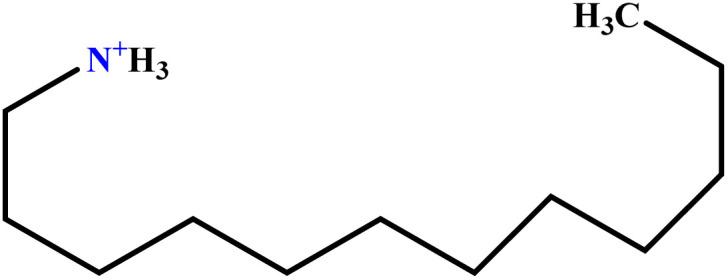	107.99	108	Validation	54
26	DMAC	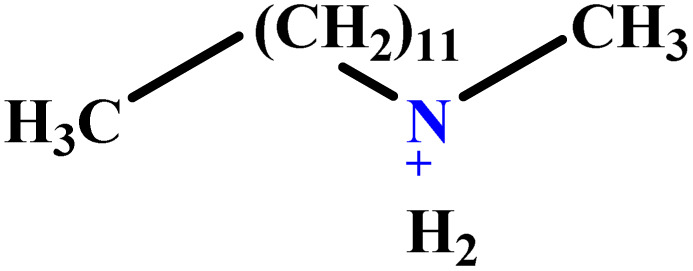	89.02	89	Test	54
27	DDMAC	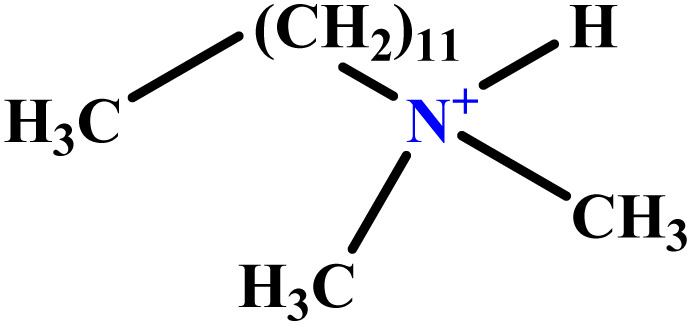	62.06	66	Training	54
28	[C_9_MIM][Br]	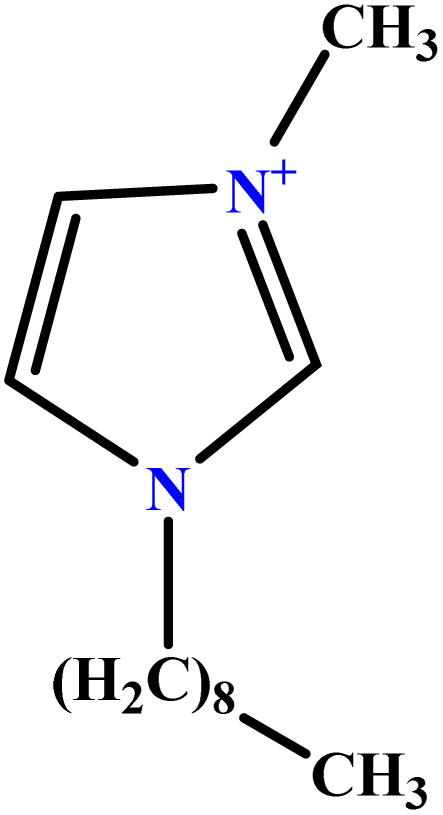	42.32	45	Training	56
29	BHDC	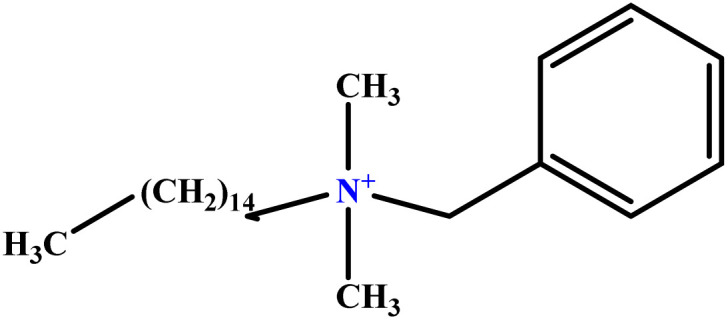	45.14	48	Training	57
30	C_12_DAB	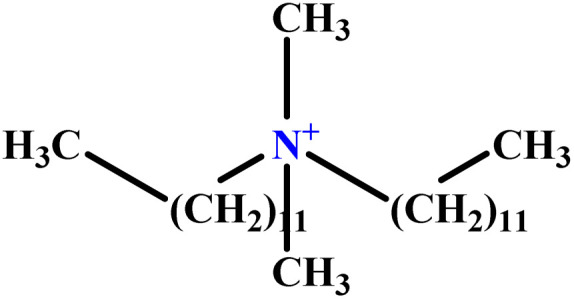	60.02	60	Test	58
31	C_16_TAB	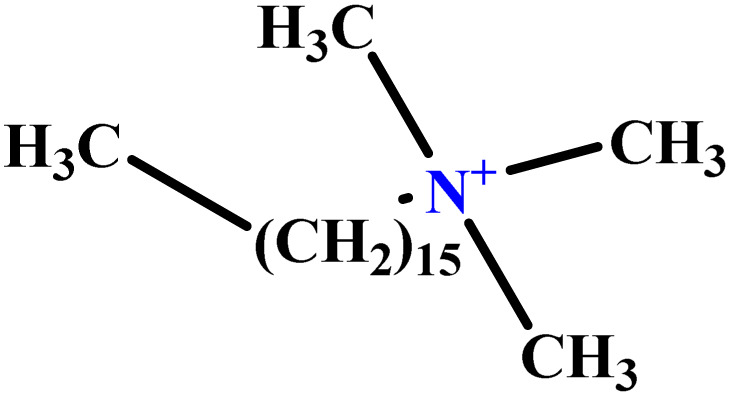	89.32	95	Training	59
32	C_14_TAB	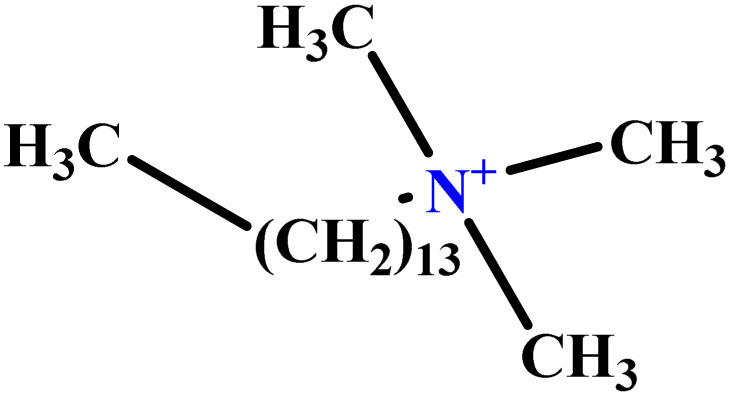	63.94	68	Training	59
33	C_12_TAB	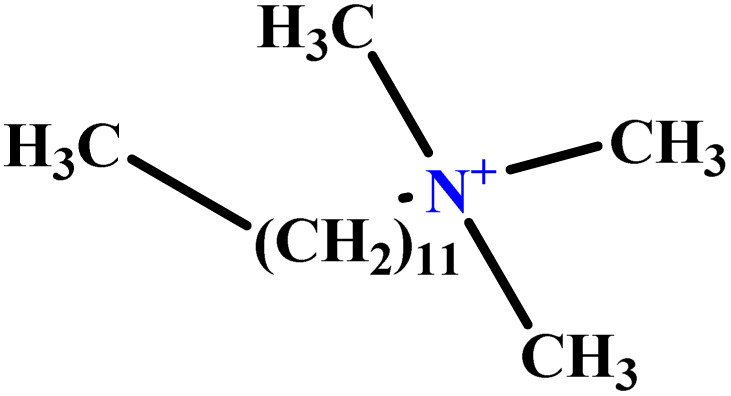	53.60	57	Training	59
34	C_10_TAB	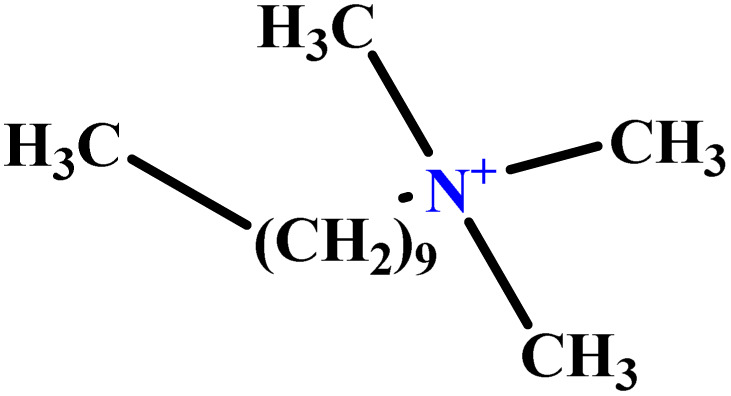	36.68	39	Training	59
35	C_6_TAB	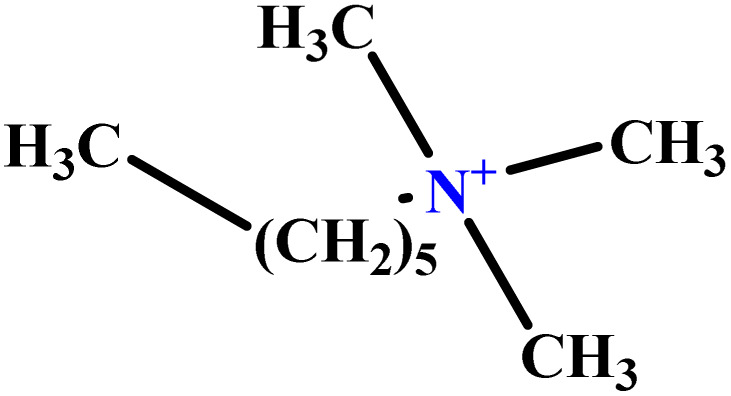	3.78	4	Training	52
36	CTAC	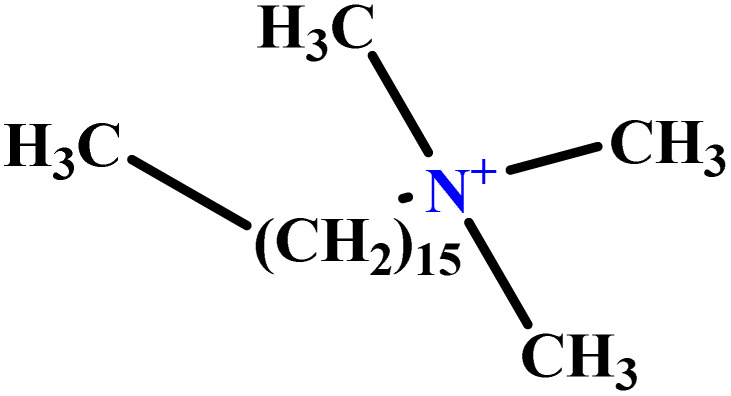	106.24	113	Training	54
37	[C_16_MIM][Br]	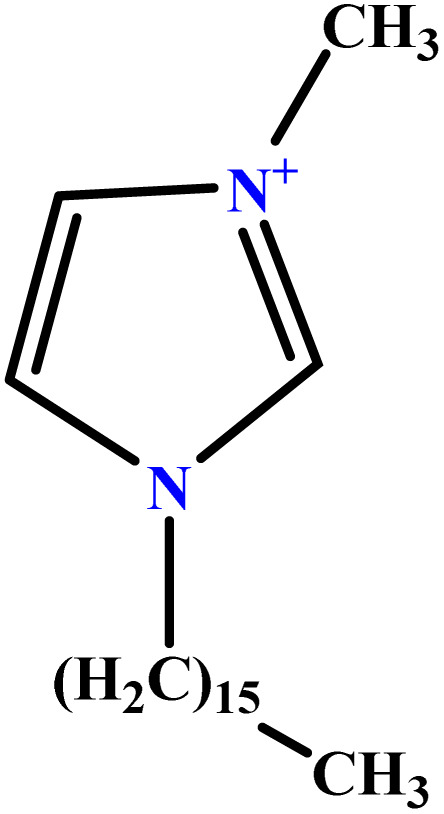	93.08	99	Training	56
38	[C_14_MIM][Br]	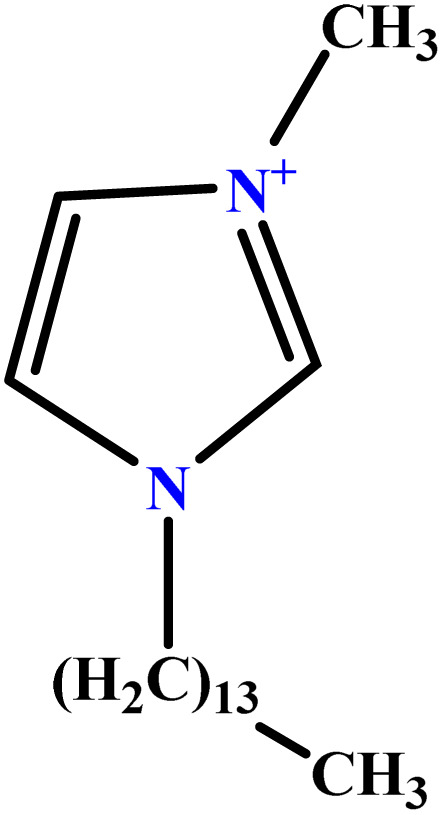	74.28	79	Training	56
39	[C_12_MIM][Br]	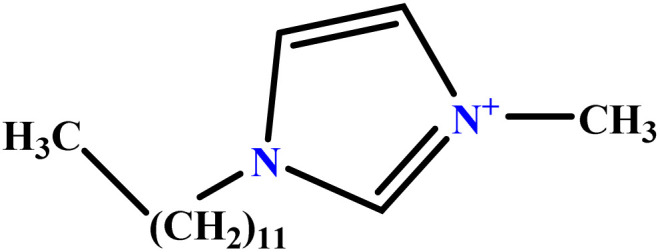	58.02	58	Test	56
40	[C_10_MIM][Br]	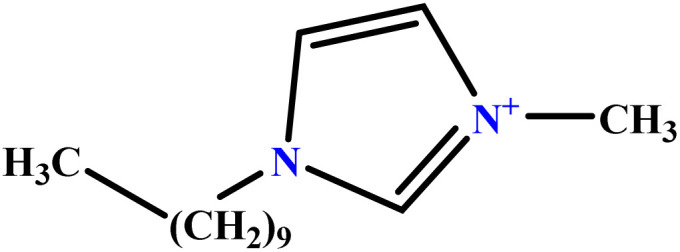	39.99	40	Validation	56
41	CPC	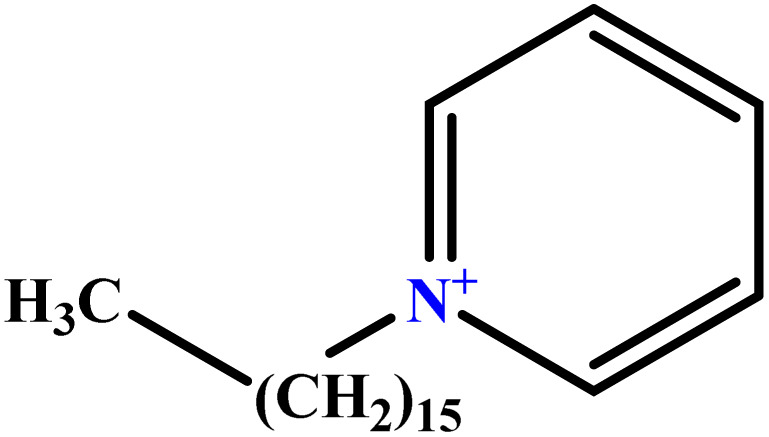	48.90	52	Training	59

### 2.1 Molecular modeling

The structure of each compound was drawn with GaussView 5.0.8 (Table 1), and optimized by the semi-empirical method, PM6, available in the Gaussian 09 software package,^60^ and the optimization goal was to achieve the structures with the lowest energy level. Due to the space limitation, the optimized structure of the molecules are shown in Table S1 (ESI).† Because the molecules were large, we preferred to use semi-empirical PM6 for optimization purposes rather than the density functional theory (DFT) as a quantum mechanical method. Indeed, PM6 can be employed for systems with thousands of atoms while retaining the benefits of the DFT calculations: they are based on a proper physical description of the molecular structure and do not depend on system-specific parameters.^61^ Moreover, the computational speed of the PM6 method is more rapid than that of DFT.

Finally, for each optimized structure, the molecular descriptors were computed using the Dragon 5.5-2007 software designed as a user-friendly software.^62^ In this software, descriptor calculations are conducted according to these simple steps: firstly, the molecular file obtained from Gaussian is loaded; secondly, the descriptors are selected; thirdly, the descriptors are computed; and fourthly, the calculated descriptors are saved. In this study, the QSAR data obtained were collected in an Excel file (see ESI† for further information). All the calculations were conducted in MATLAB, version 7 (Math Works), and the ANN was performed using the MATLAB Neural Network Toolbox.^63^

## 3. Artificial neural network

The ANNs are computer programs inspired by the human brain. They have been designed to simulate the processing information in the brain and are widely used in different branches of science such as analytical, physical, organic, inorganic chemistries, and medicinal material sciences.^43–45,64,65^ The ANNs obtain their knowledge by finding the patterns and relationships in data through experience.^66^ They are made of artificial neurons which are connected with coefficients (weights), constituting the neural structure and organized in layers.

In ANNs, each neutron possesses weighted inputs, transfer function and one output. The behavior of an ANN depends on the transfer functions of its neurons, the learning rule, and the architecture itself. The signal of the neuron is established by the weighed sum of the inputs and passed through the transfer function to create a single output of the neuron. The role of the transfer function is to introduce non-linearity to the network. The ANN algorithm is a two-step processing technique, involving training and validation steps. During training, the weights are optimized until the prediction error is minimized, and the network gains an acceptable level of accuracy. When the network is trained and tested, it can be applied for predicting the output using new input information.^67^

A variety of types of ANNS have been designed up to now, however, the majority of today's applications apply back-propagation feed-forward ANN (BPFF-ANN).^67^ This network consists of at least three layers including input, hidden and output layers. The first one is the input layer which simply serves to enter the input variables, which are the selected descriptors in this investigation. The output layer is the last one where the output variables are handled, here, the number of nodes of this layer is set to one assigning the AGGN of each surfactant. The layers between the input and output ones are called hidden layers, each of which may function independently and may transfer its results to the other one. The most crucial step in designing the ANN is optimizing the number of nodes in the hidden layer, apart from adjusting the weights, as described in the following section.

## 4. Optimization of ANN

In an ANN algorithm, there is a connector neuron between an input and a hidden layer, as well as between the neurons and the output layer, called the weight (*W*_*ij*_), which represents the “artificial synapses”.^68^ The input signals (In) are processed in the “body” of the neuron as follows:1
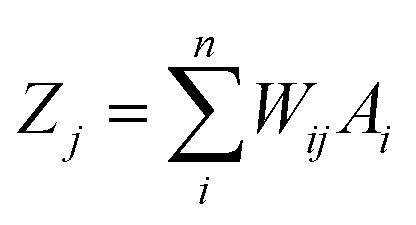
where *Z*_*j*_ and *W*_*ij*_ are the values of the *j*^th^ hidden neuron and the weight linking the *i*^th^ input neuron to the *j*^th^ hidden neuron, respectively. *A*_*j*_ is the value of the *i*^th^ input neuron, which is a normalized value of the *i*^th^ independent variable.

In ANN analysis, each variable (input or output values) is rescaled to a new range of values between −1 to +1 as follows:^69^2
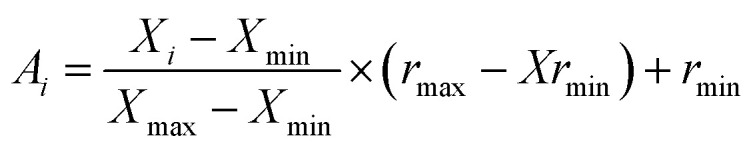
where *X*_*i*_ is *i*^th^ real variable, *A*_*i*_ is the normalized value of *X*_*i*_, *X*_min_ and *X*_max_ are the minimum and maximum values of *X*_*i*_, respectively, and *r*_min_ and *r*_max_ are attributed to the limits of the range where *X*_*i*_ should be scaled.

In the ANN algorithm, the initialization is conducted with random weights and a different initialization is done to diminish the probability of a convergence to a local minimum. The total data is divided into three sets: training, test and validation. The training set is employed to adjust the weight factors on the ANN, and the test set is used to overcome the over-fitting problem and to find the optimal number of neurons in the hidden layer. The validation set is applied to confirm the actual predictive power of the ANN.

In the BPFF-ANN algorithm, the weights change during each iteration with the aim of minimizing the difference between the actual outputs and the model predicted ones, and the change of each weight can be written as:Δ*W*_*ij*_ + *W*_*ij*_ → *W*_*ij*_

3 Δ*W*_*ij*_ = *η*(*t* − *o*)In_*i*_

where, for each sample, *t* and *o* are the target and the output value of ANN, respectively, and, *η* is the learning factor whose role is controlling the amount of weight change at each iteration. The value of *η* is usually small (*e.g.*, 0.1) and it diminishes, and would have less and less effect as the number of iterations increases.

In the ANN studies, models with fewer variables result in diminishing the complexity of the analysis, preventing overfitting/overtraining and reducing the computational time and improving the prediction power for new samples. Here, firstly, the descriptors of the surfactant molecules with zero values were omitted, and then the descriptors showing a high correlation coefficient with each other were eliminated, and finally based on stepwise regression analysis, four significant descriptors were selected for further analysis (Table 2). These variables had high correlation with the response and less correlation with each other.

**Table 2:** The selected structural descriptors for QSAR analysis

ID	Name	Description	Block
1	JGI6	Mean topological charge index of order 6	2D autocorrelations
2	H-047	H attached to C^1^(sp^3^)/C^0^(sp^2^)	Atom-centred fragments
3	Mor20p	Signal 20/weighted by atomic polarizability	3D-MoRSE descriptors
4	ESpm12x	Spectral moment 12 from edge adjacency matrix weighted by edge degrees	Edge adjacency indices

The four selected descriptors (Table 2) were applied as input neurons in the ANN modeling, and the AGGN of the surfactant molecule was considered as a neuron in the output layer. The number of hidden layers and their neurons were chosen by optimizing the model in the ANN-Matlab toolbox (Matlab *nntool*) using a BPFF-ANN algorithm. The important network parameters in the toolbox such as topology, number of data values in each classified set (training, validation and test set), and the training algorithm and its parameters are shown in Table 3.

**Table 3:** Network parameters (in the ANN-Matlab toolbox) in the QSAR analysis of the cationic surfactants

Topology	Four inputs, one output and one hidden layer with five neurons (4 × 5 × 1)
Data	Training set: 70% randomly selected observation data (29 data values)
	Test set: 15% randomly selected observation data (6 data values)
	Validation set: 15% randomly selected observation data (6 data values)
Beginning function	Log–sigmoid
Training algorithm	Levenberg–Marquardt algorithm
Loss function conditions	Minimum MSE
Stopping conditions	The network stops in one of three ways:
	Validation check > 10
	Minimum gradient < 10^−7^
	Momentum speed > 10^10^

The performance of the ANN model was evaluated based on some statistical parameters such as mean square error (MSE), square of correlation coefficient (*R*^2^), root mean square error (RMSE) introduced in the following equations:^70^4
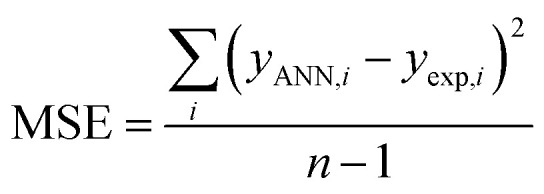
5
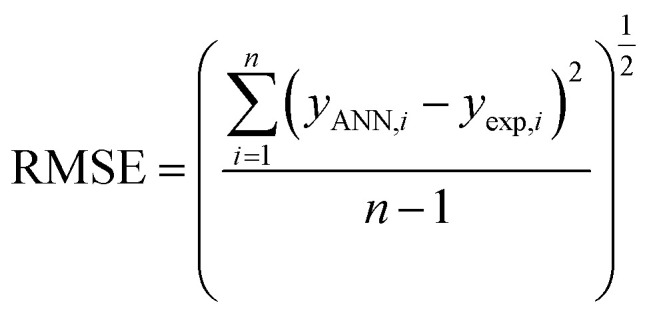
6
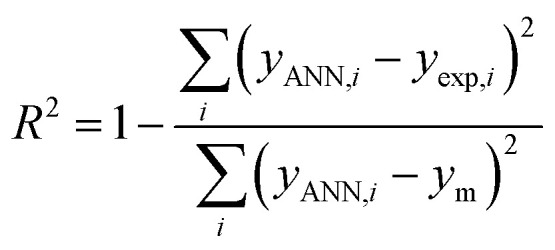
where *y*_ANN,*i*_ and *y*_exp,*i*_ are predicted, and the experimental value of AGGN for the *i*^th^ cationic surfactant molecule, respectively, *y*_m_ is the mean of *y*_exp_ in eqn (4)–(6), and, *n* is the number of molecules in each data set (training, test or validation set).

**Fig. 1:**
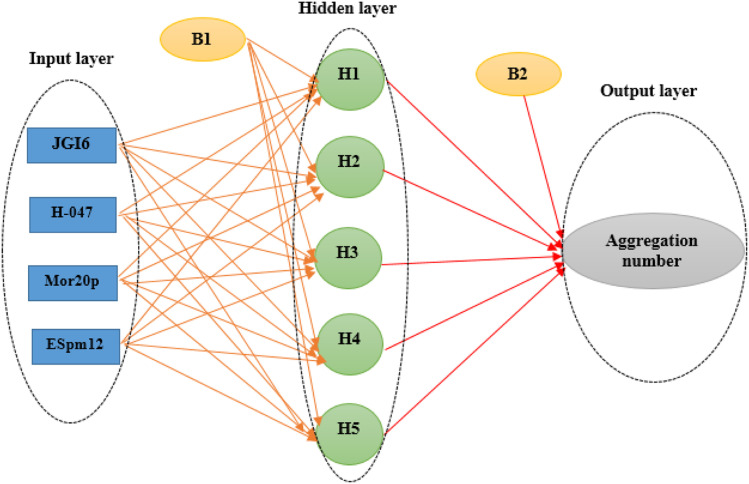
Artificial neural network architecture in QSAR studies of the cationic surfactants.

The main goal in the training step was minimizing the MSE of the test set as data which were not used during the training iterations, a fact which confirmed the ANN ability for the prediction of the new data. Here, the optimal ANN architecture was achieved according to the minimum value of the MSE and the maximum value of *R*^2^ of the test set. A network (4 × 5 × 1) was the optimal model whose topology is illustrated in Fig. 1.

**Fig. 2:**
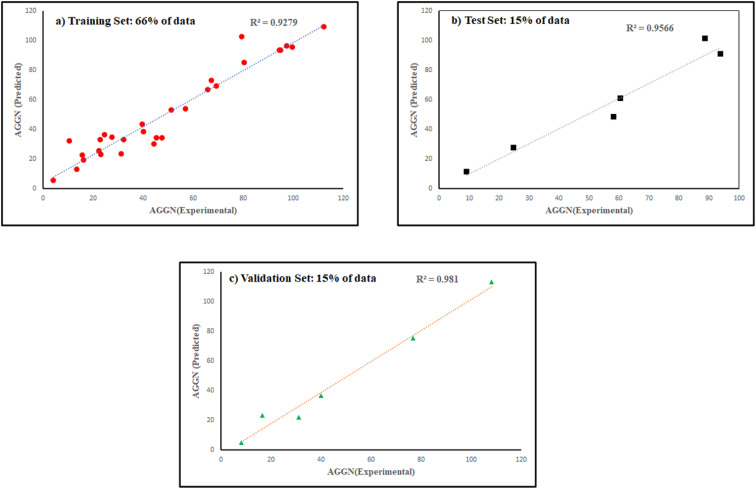
The plots of predicted AGNNs determined by ANN analysis *versus* experimental AGNNs of cationic surfactants molecules for the three data sets used in the ANN analysis.

The molecules in each data set were analyzed by the optimal ANN algorithm, and their AGGN values were estimated to clarify the prediction ability of this non-linear model. All the results were converted to the original state and plotted *versus* the corresponding experimental AGGNs as shown in Fig. 2. Table 4 shows a summary of statistical parameters such as the values of *R*^2^, MSE and RMSE for training, validation, and test sets using the ANN method. The *R*^2^ values between the experimental and predicted results reveal that the ANN model was highly efficient for the analysis of the QSAR data studied.

**Table 4:** Statistical parameters of the ANN and MLR models in the QSAR studies of cationic surfactants

Set of data	*R* ^2^		MSE		RMSE	
	ANN	MLR	ANN	MLR	ANN	MLR
Total	0.9392	0.5010	8.7070	507.1145	2.9508	22.5192
Training	0.9256	0.4578	12.4385	528.9620	3.5268	22.9992
Test	0.9526	—	4.80 × 10^−4^	—	0.0219	—
Validation	0.9762	0.6053	8.67 × 10^−5^	2.4595 × 10^3^	0.0093	49.5936

**Fig. 3:**
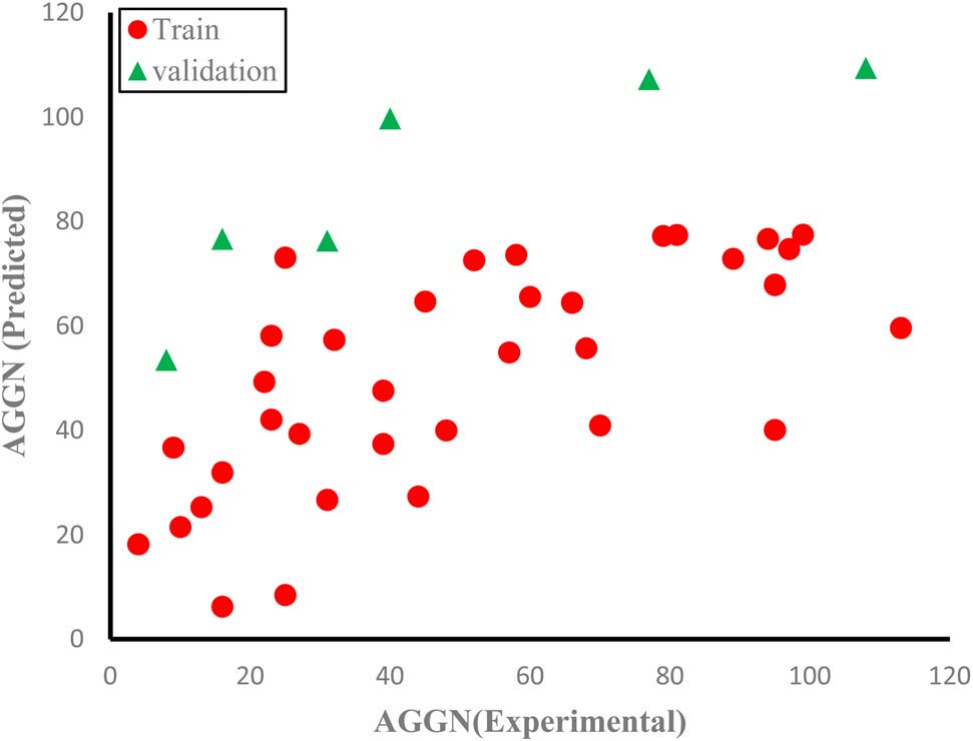
The scatterplot of predicted AGNNs by MLR analysis *versus* experimental AGNNs of cationic surfactants molecules in different data sets.

**Fig. 4:**
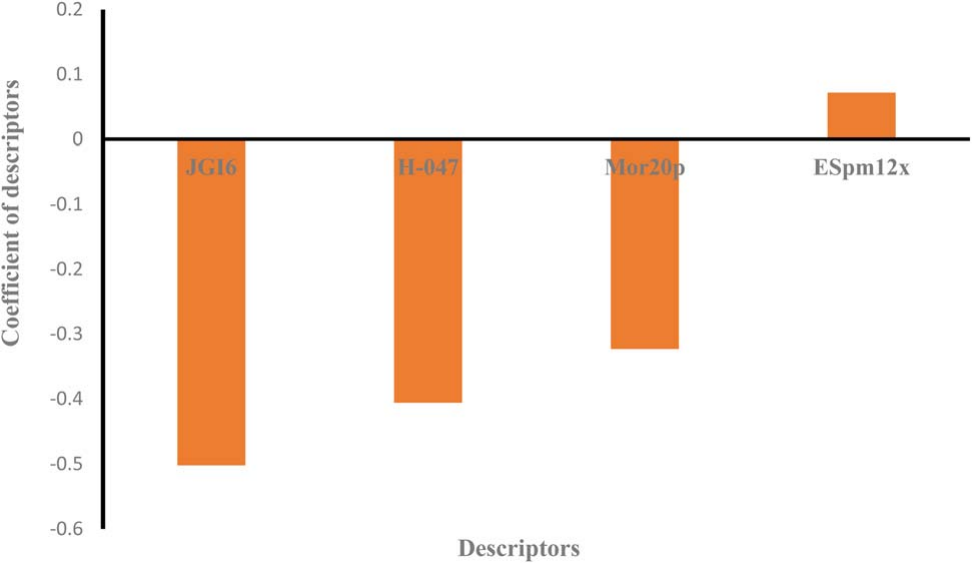
The plot of coefficients of descriptors in MLR modeling *versus* descriptors' names for QSAR study of the cationic surfactants.

Moreover, the studied data was analyzed by MLR methodology and the results were compared with the ANN strategy to reveal the necessity of employing non-linear modeling in this investigation. Fig. 4 illustrates the MLR coefficients *versus* the descriptors. Some statistical parameters of the MLR model are given in Table 4 and the correlation between the experimental and predicted results of the MLR model are shown in Fig. 3. The compared results showed that ANN is a powerful tool for detecting the relationship between the surfactant molecules and their AGGNs. This could be attributed to the non-linear relationship between the molecular structures of the surfactants and their AGGNs. To investigate this claim for each selected descriptor, the AGGN values of the surfactants molecules were plotted against the values of the descriptor, as shown in Fig. S1 (ESI).† This figure illustrates the non-linearity in this data, furthermore, Fig. S2 (ESI)† shows the non-linear relationship between the AGGNs of a set of molecules with the same hydrocarbon chain length but different polar head groups (CPC, [C_16_MIM]Br, C_16_TAB, [C_16_hpim]Br, and C_16_E_2_TAB).

## 5. Effect of input variables

The weight values in the ANN network can be employed to estimate the relative importance of each input variable on the output target using the Garson method, a numerical approach, as follows:^71^7
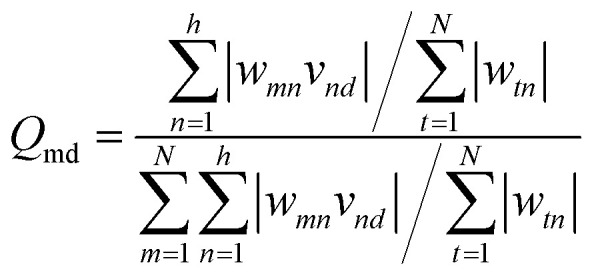
where *w*_*tn*_ is the weight between the *m*_th_ input and the *n*_th_ hidden neuron, and *v*_*nd*_ represents the weight between the *n*_th_ hidden neuron and the *d*_th_ output target.

In this study, the percentage of influence of the input variables on the AGGNs was estimated by incorporating input-hidden and hidden-output connection weights based on eqn (7), and the results are reported in Table 5. The trend of importance of the input descriptors was in the following order: H-047 > ESpm12x > JGI6> Mor20p.

**Table 5:** Effective weight matrix of the ANN modeling in the QSAR studies of cationic surfactants

Input descriptors				Hidden neurons	Hidden to output
JGI6	H-047	Mor20p	ESpm12x		
2.8220	−3.1771	−2.1059	−2.2939	H1	2.4575
1.2441	−1.3730	3.3333	0.7633	H2	−1.2424
1.7163	2.5627	−0.8406	−4.1556	H3	−4.2588
1.8402	2.8236	0.8486	−0.2216	H4	−4.9700
2.8215	8.2460	0.7830	−3.1571	H5	3.8160
22.15	38.57	16.78	22.47	Relative importance (%)	

In MLR analysis, the distribution coefficients of these descriptors were assigned by their importance. Although the importance trend in MLR analysis did not coincide with the previous trend, the sign of the coefficient gives complementary information about the descriptors. Indeed, the signs can help us interpret the relationship between the AGGNs of the molecules and the descriptors and provide more relevant information, as will be discussed next.

Descriptor H-047 belongs to atom-centered fragments (ACF) class descriptor which shows a structural fragment, H, attached to C^1^(sp^3^)/C^0^(sp), in a molecular structure, where the superscript of C denotes the formal oxidation number of the carbon atom.^72^ This oxidation number is the sum of the conventional bond orders with electronegative atoms. The fewer hydrogen atoms that are attached to sp or sp^3^ hybridized carbon atoms there are, the higher H-047 descriptor observed.^72^ As shown in Fig. 4, this descriptor has a negative effect on the AGGN as expected because fewer hydrogen atoms lead to a higher AGGN. Therefore, the H-047 descriptor recommends fewer hydrogen atoms be attached to sp or sp^3^ hybridized carbon atoms to increase the AGGN of the titled compounds. For example, here, increasing the number of hydrogen atoms attached to sp or sp^3^ hybridized carbon atoms for DMAC (−0.81 of H-047), DDMAC (−0.63 of H-047), and [C_16_MIM][Br] (−0.81 of H-047), C_16_TAB (−0.45 of H-047) molecules causes the AGGN to decrease from 89 to 66, and 99 to 95, respectively.

ESpm12x is the spectral moment of the edge-weighted adjacency matrix which is represented by the structural fragments present in the molecules.^73^ This descriptor has been widely used for the interpretation of physical and physicochemical properties of alkanes and has presented powerful significant models from the statistical point of view. Indeed, the molecules with higher ESpm12x values belong to the higher length of the hydrocarbon chain.^75^ As shown in Fig. 4, this descriptor has a positive effect on the AGGN property, indicating that the ESpm12x is directly related to the AGGN.

JGI6 is a mean topological charge index of order 6 which can assess both the charge transfer between pairs of atoms and the global charge transfer in a molecule.^74^ This descriptor represents the total charge transfer between atoms at a topological distance of 6 which are closely related to substitutions at the peripheral molecular sites, and molecular polarity. In a molecule, the higher the charge transfer is, the higher the JGI6 value observed.^74^ In this study, the JGI6 descriptor showed a negative impact on AGGN and it is expected that a smaller AGGN will be observed for a molecule with higher polarity and, in turn, a higher charge transfer. For example, both C_16_TAB (−0.44 of JGI6) and CPC (−0.75 of JGI6) molecules have the same hydrocarbon chain length but in CPC, due to the resonance and charge distribution on the molecule surface, the charge transfer is higher and the molecule has a smaller AGGN.

The Mor20p descriptor expresses the 3D structure of a molecule and encodes information about the polarizability and is similar to the JGI6 descriptor, but the Mor20p value of a molecule is directly related to the polarizability of the molecule. As discussed, previously, the increase in polarizability of a molecule results in the decrease in AGGN. For example, C_16_TAB (−1 of Mor20p) and CPC (−0.3694 of Mor20p) molecules have the same hydrocarbon chain length but a different polar head group and the Mor20p of CPC molecule is higher than that of C_16_TAB, and as expected, its AGGN is lower due to its higher polarity.^75^

Overall, it can be concluded that the decreased values for H-047, JGI6 and Mor20p, together with the increased value for the ESpm12x descriptor will provide higher values for the AGGN property of the studied cationic surfactants.

## 6. Conclusions

In this study, the QSAR study was performed on some cationic surfactants to correlate the molecular structure of the surfactants with their AGGNs. Among more than 3000 molecular descriptors that were considered in generating the QSAR model, four descriptors resulted in a statistically significant model.

The QSAR data was analyzed based on both linear (MLR) and non-linear (ANN) modelling techniques and the results of these methods were compared statistically. A higher *R*^2^ and a lower RMSE of the ANN method were achieved, a fact which supports the efficiency of ANN in detecting relationships between surfactant molecules and their AGGNs with a high predictive power.

In summary, the QSAR-ANN was proposed as a promising technique to predict the AGGNs of surfactants and to obtain extract maximal information about the surfactant systems with minimal experimental effort.

## Conflicts of interest

There are no conflicts of interest to declare.

## Supplementary Material

RA-012-D2RA06064G-s001

RA-012-D2RA06064G-s002
