# Supplementary material for: Predicting the aggregation number of cationic surfactants based on ANN-QSAR modeling approaches: understanding the impact of molecular descriptors on aggregation numbers
Source: RSC Adv. 2022 Nov 24;12(52):33666–78. doi: 10.1039/d2ra06064g (PMC9685374; doi:10.1039/d2ra06064g)
Supplement: RA-012-D2RA06064G-s002 [file RA-012-D2RA06064G-s002.pdf]

Electronic Supplementary Material (ESI) for RSC Advances.
This journal is © The Royal Society of Chemistry 2022

Table S1. The Optimized Molecular structure and AGGN value of each cationic surfactant in ANN-QSAR studies

No.	Symbol	molecular structure	Pred. AGGN	Exp. AGGN	Set of data	Ref.
1	m-X-3	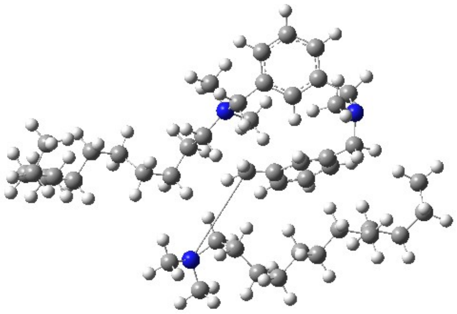	15.06	16	Training	40
2	EO-2	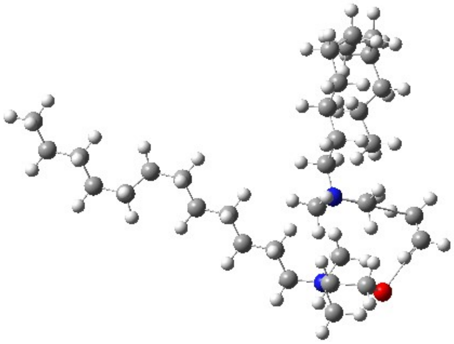	30.99	31	Validation	40
3	t-B-2	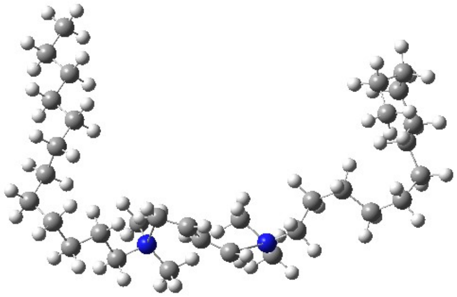	29.16	31	Training	40
4	o-X-2	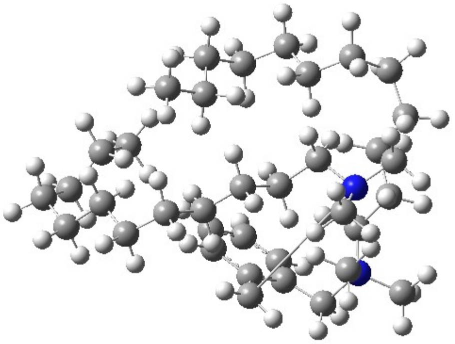	25.02	25	Test	40

5	BDDAC<br>$C_{21}H_{38}ClN$	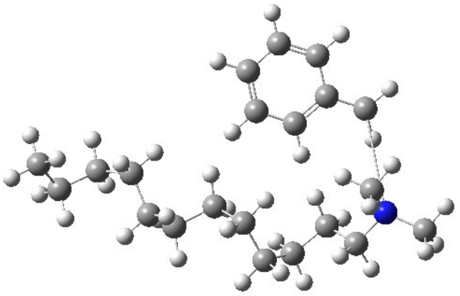	25.40	27	Training	40
6	[BisDec(MIM)$_2$]<br>[2Br]	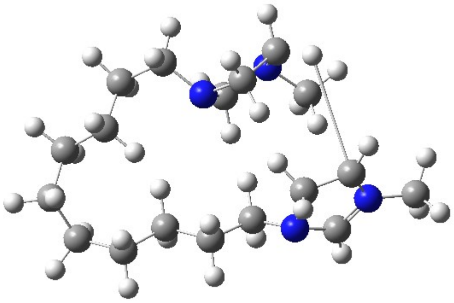	65.82	70	Training	41
7	[BisOct(MIM)$_2$]<br>[2Br]	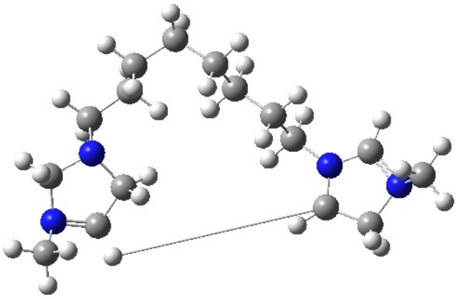	36.68	39	Training	41
8	[BisHex(MIM)$_2$]<br>[2Br]	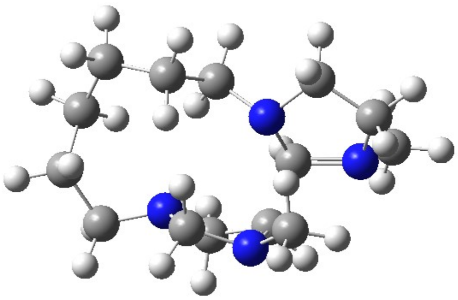	15.99	16	Validation	41

9	ValC3LS	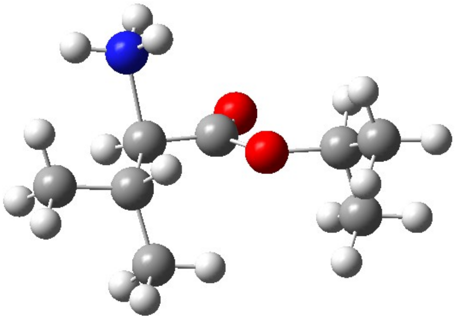	76.99	77	Validation	42
10	ProC3LS	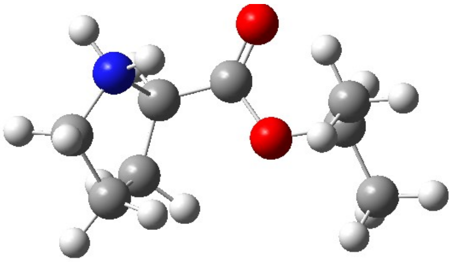	41.38	44	Training	42
11	AlaC3LS	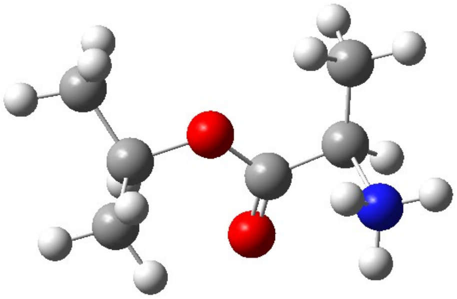	76.16	81	Training	42
12	GlyC3LS	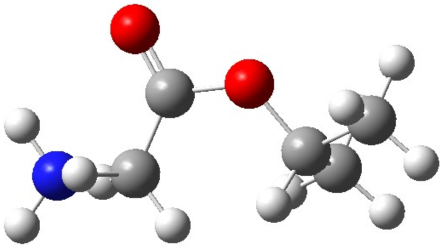	94.02	94	Test	42
13	$[C_{16}hpim]Br$	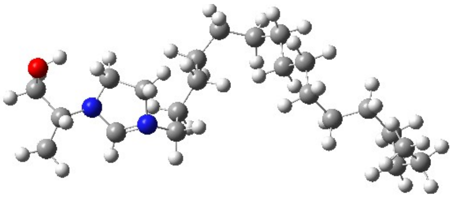	23.52	25	Training	43

14	L-UCPB	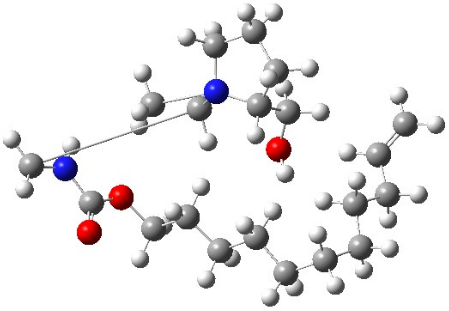	89.32	95	Training	44
15	LUCLB	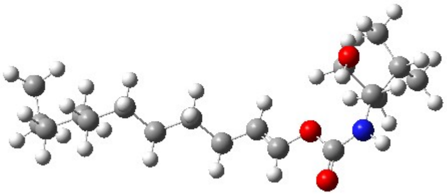	91.20	97	Training	44
16	[C_8mim][Cl]	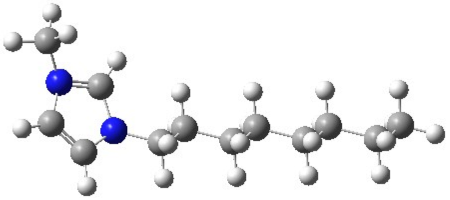	21.64	23	Training	45
17	[C_4mpy][Cl]	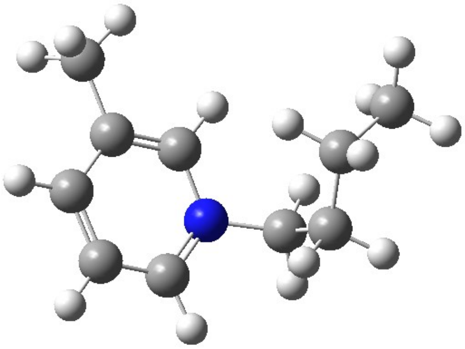	12.24	13	Training	45
18	[C_4mim][Cl]	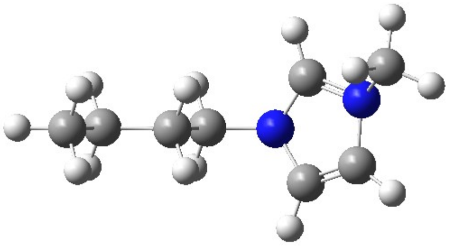	7.99	8	Validation	45

19	PH	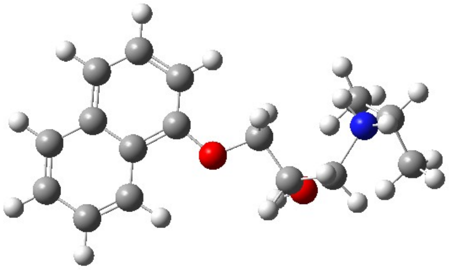	9.41	10	Training	46
20	$C_{16}E_2TAB$	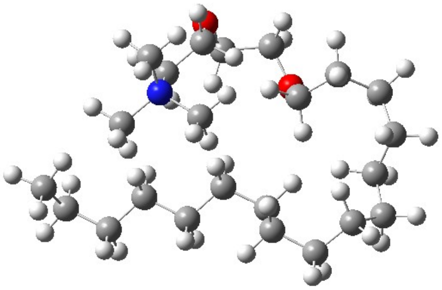	30.10	32	Training	47
21	$C_{14}E_2TAB$	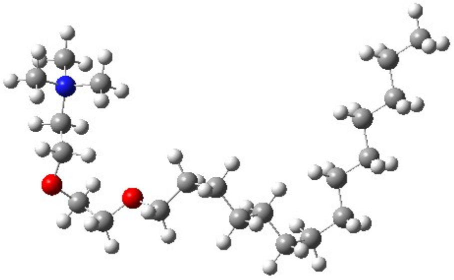	21.64	23	Training	47
22	$C_{10}E_2TAB$	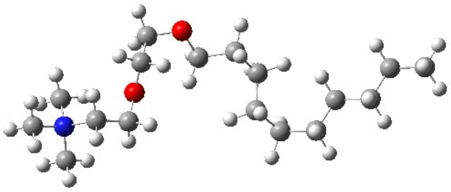	9.02	9	Test	47
23	$C_{12}E_3TAB$	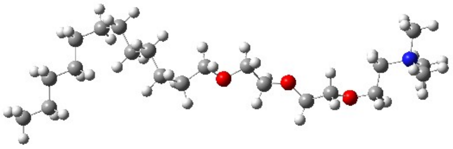	15.06	16	Training	47

24	$C_{12}E_2TAB$	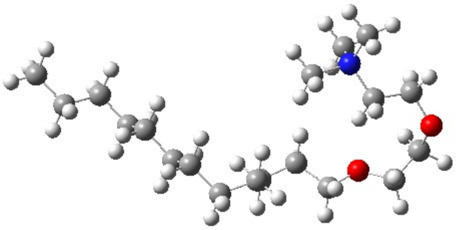	20.70	22	Training	47
25	DAC	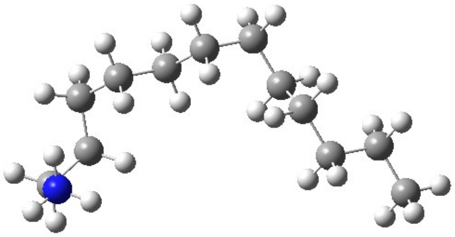	107.99	108	Validation	48
26	DMAC	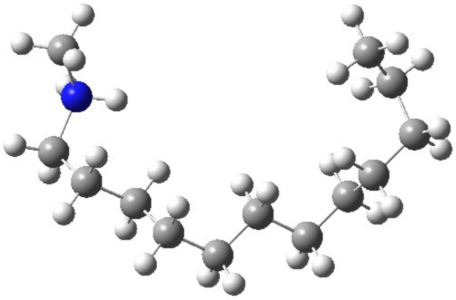	89.02	89	Test	48
27	DDMAC	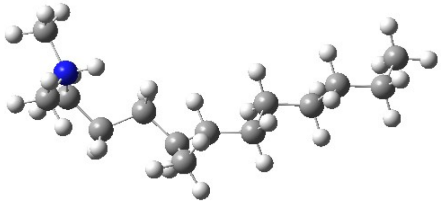	62.06	66	Training	48
28	$[C_9MIM][Br]$	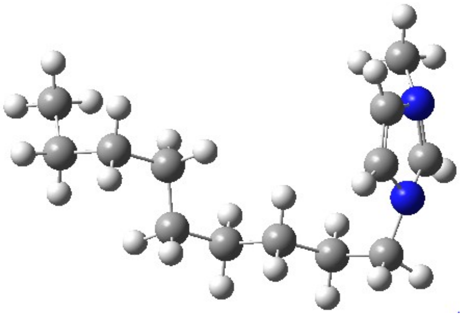	42.32	45	Training	50

29	BHDC	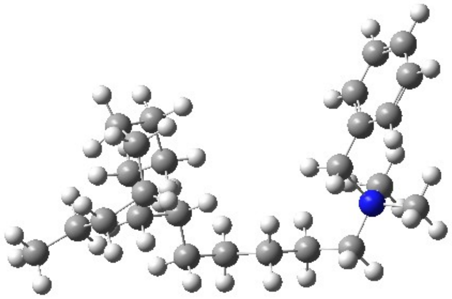	45.14	48	Training	51
30	$C_{12}DAB$	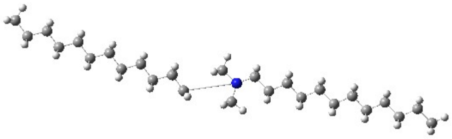	60.02	60	Test	52
31	$C_{16}TAB$	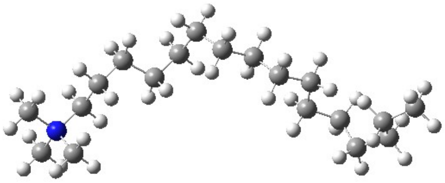	89.32	95	Training	53
32	$C_{14}TAB$	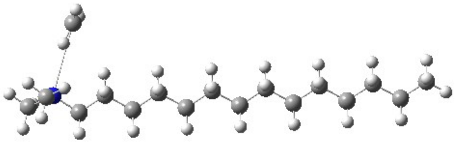	63.94	68	Training	52
**33**	$C_{12}TAB$	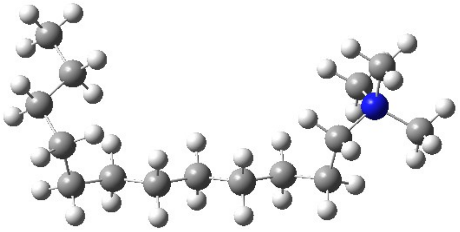	53.60	57	Training	52
**34**	$C_{10}TAB$	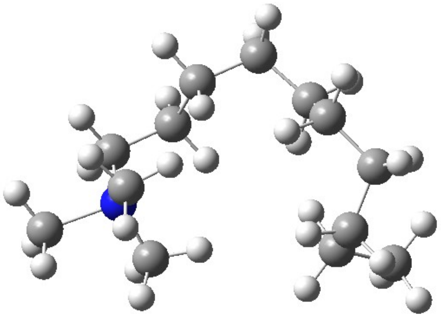	36.68	39	Training	52

**35**	C_6TAB	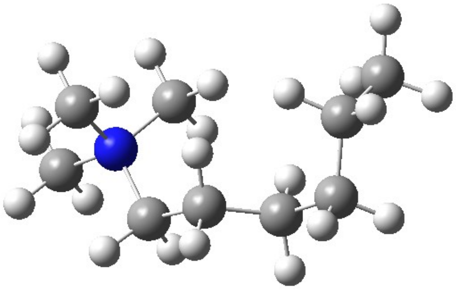	3.78	4	Training	46
**36**	CTAC	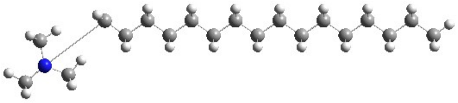	106.24	113	Training	48
37	[C_{16}MIM][Br]	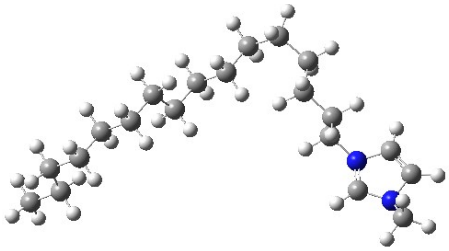	93.08	99	Training	50
38	[C_{14}MIM][Br]	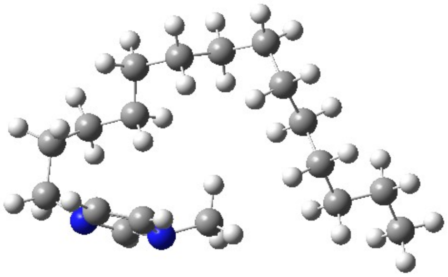	74.28	79	Training	50
39	[C_{12}MIM][Br]	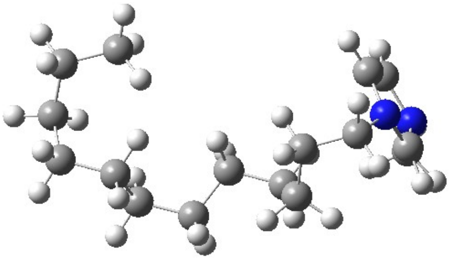	58.02	58	Test	50
40	[C_{10}MIM][Br]	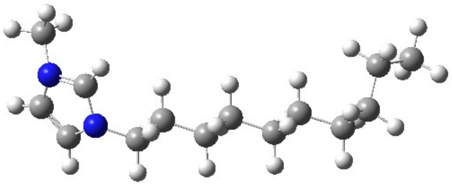	39.99	40	Validation	50

41	CPC	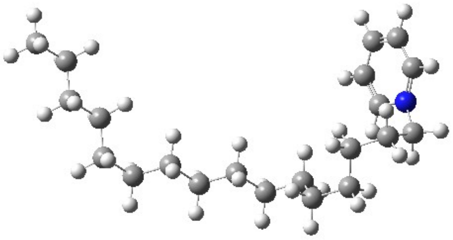	48.90	52	Training	53

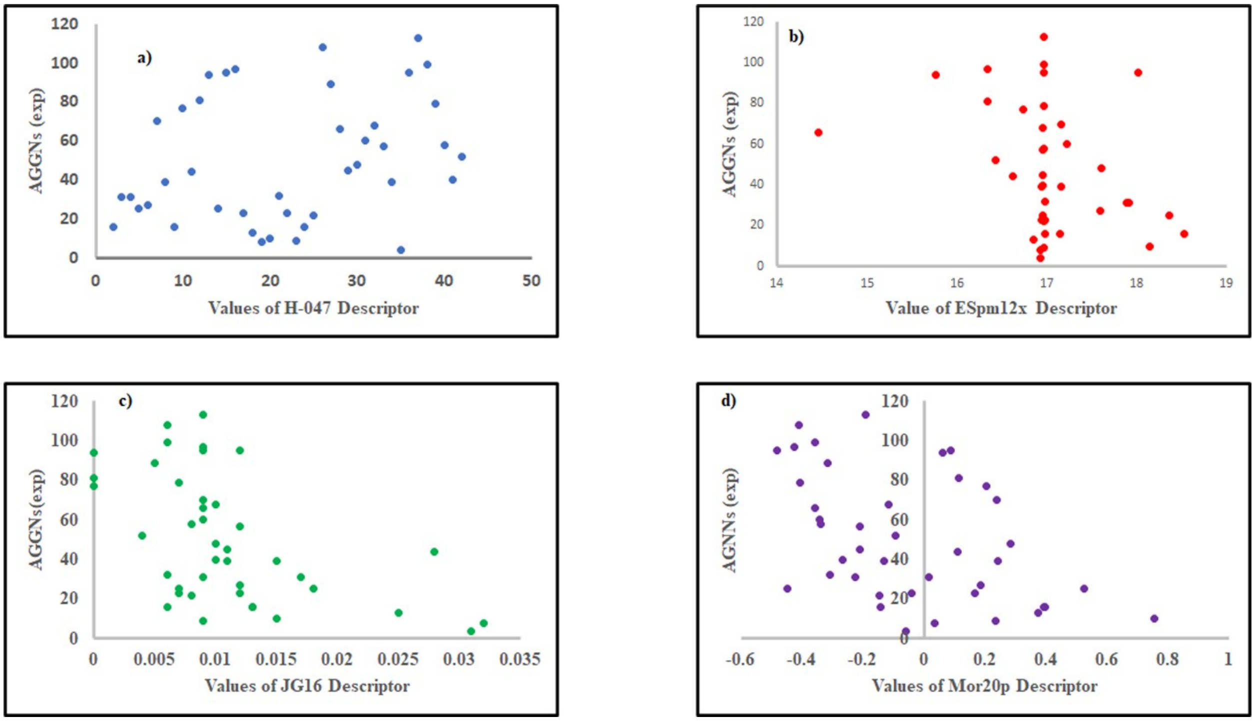

Fig S1. The plots of AGGN values of total data set versus the values of each selected descriptor

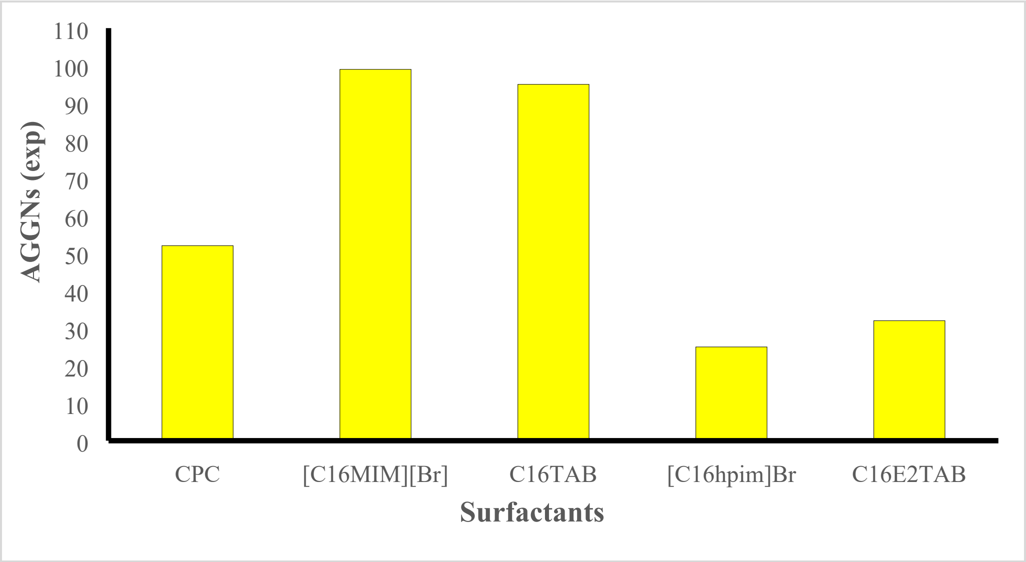

Fig S2. The plots of AGGNs values of some cationic surfactants with the same hydrocarbon chain length but different polar head groups
